# Dental Wreath

**Published:** 1857-03

**Authors:** 


### DENTAL WREATH.

The beautiful poem which we published in our last issue, seems to elicit but one opinion from our readers. As far as we have heard, it is universally commended, and indeed it could scarcely be otherwise. "Dentologia" and its fellow must yield the palm to the "Dental Wreath;" but it will not hurt the feelings of the gallant Dr. Brown to be surpassed by a lady.

